# Social Licence to Operate: What Can Equestrian Sports Learn from Other Industries?

**DOI:** 10.3390/ani12151987

**Published:** 2022-08-05

**Authors:** Janet Douglas, Roly Owers, Madeleine L. H. Campbell

**Affiliations:** 1 World Horse Welfare, Anne Colvin House, Snetterton, Norwich NR16 2LR, UK; 2 School of Veterinary Medicine and Science, University of Nottingham, Sutton Bonington, Nottingham LE12 5RD, UK

**Keywords:** equestrian sport, equestrianism, equine ethics, equine welfare, social licence to operate, trust

## Abstract

**Simple Summary:**

Most societies regulate human activities using laws that state clearly what is, and is not, legally permissible. However, there is a second layer of permission that is granted—or revoked—by the public. This second layer is known as a ‘social licence to operate’ (SLO), and it represents an intangible, implicit agreement between the public and an industry or group. The public may approve of an activity, in which case it can proceed with minimal formalised restrictions, or it may disapprove, and this may herald legal restrictions, or even an outright ban. This review discusses the concept of SLO in relation to equestrianism. Experience from other industries suggests that, to maintain its SLO, equestrianism should take an ethics-based, proactive, progressive, and holistic approach to the protection of equine welfare, and should establish the trust of all stakeholders, including the public. Trust will only ensue if society is confident that equestrianism operates transparently, that its leaders and practitioners are credible, legitimate, and competent, and that its practice reflects society’s values. Earning and maintaining this status will undoubtedly require substantial effort and funding—inputs that should be regarded as an investment in the future of the sport.

**Abstract:**

The concept of ‘social licence to operate’ (SLO) is relevant to all animal-use activities. An SLO is an intangible, implicit agreement between the public and an industry/group. Its existence allows that industry/group to pursue its activities with minimal formalised restrictions because such activities have widespread societal approval. In contrast, the imposition of legal restrictions—or even an outright ban—reflect qualified or lack of public support for an activity. This review discusses current threats to equestrianism’s SLO and suggests actions that those across the equine sector need to take to justify the continuation of the SLO. The most important of these is earning the trust of all stakeholders, including the public. Trust requires transparency of operations, establishment and communication of shared values, and demonstration of competence. These attributes can only be gained by taking an ethics-based, proactive, progressive, and holistic approach to the protection of equine welfare. Animal-use activities that have faced challenges to their SLO have achieved variable success in re-establishing the approval of society, and equestrianism can learn from the experience of these groups as it maps its future. The associated effort and cost should be regarded as an investment in the future of the sport.

## 1. Introduction

The concept of ‘social licence to operate’ (SLO) first arose in 1997 in relation to mining [1] and has since been extended to other natural resource management industries such as fishing, forestry, and energy production [2]. The concept is also relevant to animal-use industries and activities, including dairy and sheep farming, wildlife use, zoos, hunting [2], circuses [3], marine mammal parks [3], and equestrianism [2,4,5,6,7].

**Figure 1:**
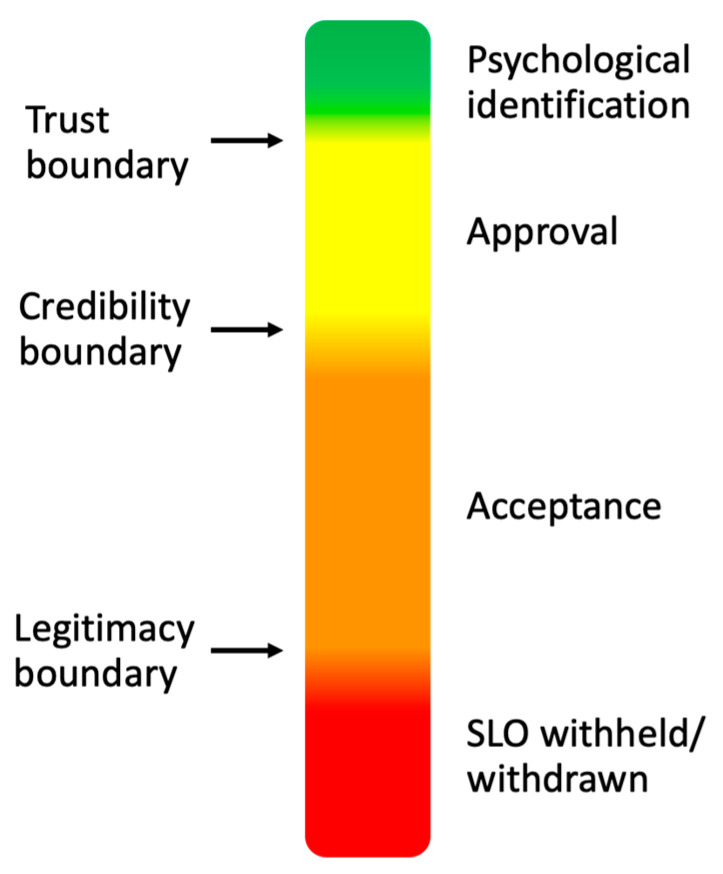
Social licence to operate. An industry’s social licence to operate is an intangible and somewhat fluid agreement between the public and those in the industry. It can swing between psychological identification of society with those engaged in the industry/activity and rejection of the activity. The point on this continuum at which the social licence sits is largely under the control of the industry itself. Graphic: After Thomson and Boutilier (2011) [13].

A social licence is said to exist when an industry or activity has the ongoing acceptance or approval of society [8], and it allows industries ‘the privilege of operating with minimal formalised restrictions’ [9]. Social licence is not an ‘all or nothing’ phenomenon, and public acceptance of an activity can sit at any point on a continuum from psychological identification with the activity to outright rejection [8] (Figure 1). It is important to recognise that the legality of an activity or industry and the state of its social licence are entirely separate entities: the former is explicit and is granted by government, whereas the latter exists only as an intangible, implicit, and somewhat fluid agreement between the public and those who engage in the activity [4,10]. However, as we discuss later, if the public rejects an industry or activity, legal restrictions are likely to follow.

In this review, we examine the relevance of the social licence concept to equestrianism, an endeavour that is typically defined as ‘the art or practice of riding a horse’ [11]. However, for the purposes of this review, we are defining equestrianism as ‘any use of horses by humans for competition, leisure, entertainment, or companionship.’ Working equids [12] are specifically excluded from our definition. The review also discusses current threats to equestrianism’s SLO and—based on the experience of other industries—suggests actions that those within the sport can take to preserve its social licence. The instigation of these actions would not only help to preserve the public acceptability of equestrian sports, but would also be of clear benefit to horses’ welfare and quality of life. Concepts that require consideration include ethics, investment in research, a focus on horses’ mental, physical, and social wellbeing, and education of all stakeholders.

## 2. How Is the Social Licence Concept Relevant to Equestrianism?

No industry or activity is exempt from the power exerted by its social licence. Public opinion can swing against any endeavour. For example, loss of social licence has resulted in closure of natural resource management industries [14]. Societal concerns have also had a serious impact on animal-use activities, including equestrianism. Examples from across a range of jurisdictions include loss of self-regulation, which has affected greyhound racing [4,15], obstructive activity by private companies (trophy hunting) [2,16], loss of sponsorship (horse racing and three-day eventing) [17,18], reduced profitability (various animal-use industries, including kangaroo harvesting) [2], and regulatory bans (greyhound racing, horse racing, and hunting of large carnivores) [2,16,19,20,21].

In 2016, the author of an investigation into greyhound racing in New South Wales, Australia, noted that “organised sports … have a ‘social licence’ to operate only as long as they perform in accordance with public expectations” [15]. This report concluded that greyhound racing had lost its social licence [15], leading directly to a ban on the sport in New South Wales and the Australian Capital Territory [22]. The ban was subsequently overturned in New South Wales, but the sport remains heavily regulated in this state [23]. Greyhound racing is also illegal in the majority of states within the United States [24,25]. These bans illustrate how the values of society can shape government policy [16]: what may start as negative media and loss of public trust can escalate into loss of political support, revised legislation, and, ultimately, a regulatory ban on the industry or activity in question [2,4,16].

Despite minority opinions to the contrary (e.g., from those opposing horseracing), the social licences of multiple branches of equestrianism, both national and international, have to date been upheld by the majority view in most societies. Such SLOs are underwritten by ongoing political support [26] and mainstream media coverage that is largely positive. However, the status quo should not be taken for granted, and current threats to equestrianism’s SLO cannot be ignored. Negative media is already evident in various branches of equestrian sports [27,28,29,30,31], and well-funded, well organised opposition to equestrianism exists [32,33,34,35]. In some jurisdictions, public pressure has led to the introduction of external regulation (e.g., the Horseracing Integrity and Safety Authority in the United States) [36], and the loss of equestrian sports based on welfare concerns is not unprecedented: in 1997, jump racing was banned in New South Wales, Australia, under the state’s Prevention of Cruelty to Animals Act [21], and in 2020, the National Federation of the United Arab Emirates’ membership of the International Equestrian Federation (FEI) was suspended over welfare concerns and non-compliance with FEI rules in endurance competitions [37]. Moreover, involvement of the French National Assembly [38] in recommendations for equine welfare at the 2024 Olympic Games shows parliamentary interest in a sport that, until recently, has been largely self-regulating.

Many of the examples above focus on one equestrian sport: horse racing. However, the experience of other industries shows that suboptimal practice in one branch of an industry may impact upon the SLO for other branches [14], and that the adverse effects of an event that attracts public condemnation may be felt globally. Recent examples involving animals include the killing of Cecil the lion, which appears to have accelerated changes to trophy hunting laws, and poor riding in the modern pentathlon at the Tokyo Olympics, the repercussions of which reverberated throughout the horse world and beyond [16,39,40]. Therefore, no equestrian sport in any country can safely assume that its SLO will remain unthreatened, since the actions of a few can affect the futures of many [14].

## 3. What Underlies the Threat to Equestrianism’s SLO?

In natural resource management industries, SLO loss is usually related to practices that cause environmental or social harm [4]. In contrast, in animal-use activities, SLO loss is largely based on public concerns—real or perceived—about the safeguarding of animal welfare [2]. These concerns are currently being voiced about many aspects of equestrianism, including racing, dressage, show jumping, eventing, endurance, modern pentathlon, and showing of Tennessee Walkers [27,29,40,41,42,43,44,45,46,47].

Public disquiet about animal welfare has grown over past decades. This change—much of which is likely to enhance animals’ quality of life—has been fuelled by a population that is increasingly urbanised [2,48] and that expects a more compassionate and ethics-based approach to the welfare of animals used in recreation than was previously the case [49]. For animals, such as horses, that belong to a social species, this includes providing the opportunity to engage in bonding activities such as allogrooming with familiar conspecifics [50].

The evolution of the public’s views on animal welfare is illustrated by the altered attitude regarding animal-use activities that were once deemed socially acceptable, including the use of animals in circuses, marine mammals in aquaria, caged animals in zoos, the hunting of wildlife, and dog fighting [3,16,48,49,51]. The rise in vegetarianism and veganism in many societies is also partially based on animal welfare concerns [52].

This wave of change in attitudes towards animal welfare is fuelled by advances in technology and shifts in how society operates. The use of hidden cameras [2] and the almost ubiquitous presence of mobile phones capable of taking high quality photographs and videos has greatly increased the visibility of ‘backstage’ practices [53,54]. In the absence of context, still photographs and short videos may appear to show malpractice where none exists. However, this argument can also be used in an attempt to disguise the reality of the events captured [27]. The growth of the internet and the rising use of social media mean that such images can be disseminated instantly and widely. As a consequence, almost nothing is hidden from public view and the potential for negative reports on social media to lead to rapid changes in public perception and government policy—as has happened previously in relation to trophy hunting of wild carnivores [16]—is ever-present. In addition, it is now easier for groups that oppose the use of animals in sports to attract funding [55].

Public attitudes about animal use are also affected by advances in scientific knowledge. This is underpinned by the growing recognition that animals are sentient creatures [56,57] for whom physical, mental, and social wellbeing are important [50] and by the growth of animal welfare as an established science [58]. In addition, greater emphasis among researchers on making science available and accessible to a non-scientific audience [59,60] has increased the ease with which scientific findings can be disseminated among both the academic community and the wider public [61]. It is against this backdrop of changing public attitudes, technological advances, and scientific progress that animal-use industries must pay attention to the maintenance of their social licences—licences that are under threat of erosion in the current climate [2].

## 4. What Have the Sports’ Regulatory Bodies done to Date to Maintain Equestrianism’s Social Licence?

Rules to protect the welfare of horses have long been a part of equestrian sports [62]. However, in tandem with changes in public attitudes towards animal welfare, many of those within equestrianism have also altered their views. These evolving attitudes are reflected in changes to the rules of competition and, in some cases, the introduction of overarching equine welfare strategies and recommendations [63,64,65,66,67,68,69,70,71,72]. Initiatives that aim to promote best practice have also been implemented in a number of disciplines, including awards for best body condition [73], best condition in endurance competitions [74], and best shoeing [75]. Other recently introduced measures that aim to promote the positive protection of equine safety and welfare include improved provision for the rehoming and retraining of retired racehorses [5,76], pre-race ‘suitability to race’ examinations [77], the use of deformable and frangible devices in cross country fences [78,79], alterations to the design and placement of hurdles and steeplechase fences [77,80,81,82,83], advances in the safety training of officials [79], evolution of the rules relating to types and fitting of tack [84,85] and horse falls [79,86], generation of falls and safety databases [79,87], and improved post-exercise cooling protocols [83,88]. Changes to the rules for equestrian competition at the Paris Olympics (2024) have also been recommended [38].

Rules and sanctions have also been developed to combat a range of practices that may negatively affect equine physical and/or psychological welfare. These include rules about trimming of vibrissae [89,90,91], excessive rider weight [91,92], the presence of blood on the horse’s body or in its mouth [93], abusive training methods [67], illicit use of medication [67], use of the whip [4,94,95,96,97,98], and changes in limb sensitivity [89,99].

Advances in the protection of horse welfare made by individuals and equestrian sporting organisations notwithstanding, there remain substantial challenges to equestrianism’s SLO. The issues that pose the greatest risk are those that are publicly visible (e.g., injury during competition) or that are brought to the public’s attention by activists, whistleblowers, and journalists, some of whom are involved in the sport [27,29,40,41,42,43,44,45,46,47]. However, it is arguable that these are just the tip of the iceberg, and that many less visible welfare issues—which may, over time, enter mainstream public awareness—have a greater impact on horses’ quality of life [100]. These include the approximately 23 h/day for which many horses are stabled [100]. In this context, the relative absence, in most jurisdictions, of regulations relating to horses’ ‘down time’ compared with the plethora of regulations relating to their experience during competition is notable.

## 5. Positive Actions to Maintain Social Licence: What Can Equestrianism Learn from Other Industries? 

The threats to equestrianism’s social licence are well recognised by those leading the sport [101,102]. This is a major step in the direction of positive change, since denial of the problem is a key contributor to the demise of an industry [48]. However, the strategies that equestrianism adopts to address concerns about the validity of its social licence are likely to dictate the future of the sport [100]. Observations from other industries may be relevant in this regard.

Experience from the mining industry suggests that success in SLO retention/repair is best achieved through a number of simultaneous strategies, all of which require the industry to be proactive. In this context, proactivity involves taking ownership of issues and embracing reform [2]. When combined with the transparency of operations, this approach has been successful in both protecting and repairing an industry’s SLO [2]. This contrasts with a reactive approach to social licence problems which essentially involves denying that there is an issue and relying on positive public messaging for reputational repair. However, the public can distinguish between serious, science-based attempts to improve animal welfare and unsubstantiated positive messaging [2], and a reactive approach, although potentially successful in the short term, is unlikely to be effective in the long run [2,48]. In addition, the longer an issue remains unresolved, the more difficult it is to change public sentiment, and the more likely is the imposition of legislation [48]; and legislation, once in place, is rarely revoked [9]. Early, proactive engagement with issues is therefore recommended.

**Figure 2:**
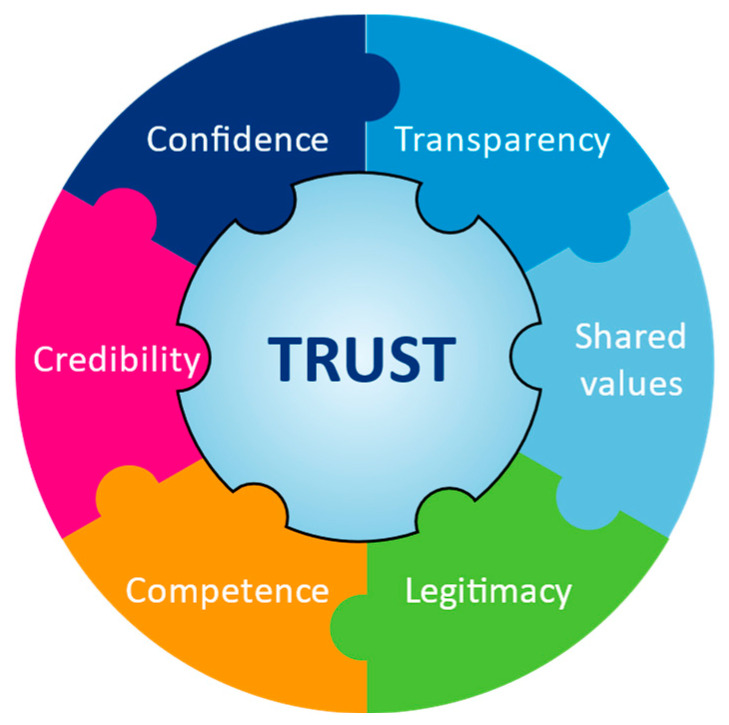
Parameters that underpin trust. Society’s trust in an industry or activity is underpinned by a range of factors, including confidence in the transparency of the industry’s operations, the credibility, legitimacy, and competence of its leaders and practitioners, and the relevance to society of the values that it espouses.

In terms of priorities, establishing public trust is key to improving the status of an industry’s SLO [2,4,14,48,103] (Figure 2). In this context, trust implies confidence that the industry will act with integrity and ‘do what’s right’ [103,104]. When faced with a negative slide in their social licence, some industries assume that they have an image problem when in fact, their problem is lack of public trust [9]. This is an important distinction because, although image and trust are related, a problem with each of these concepts requires a different response [9]. Stakeholder research can be extremely informative in this regard and can guide an industry towards beneficial change [9].

Data from the food industry suggest that establishment of trust is based primarily on how much confidence people have in that industry to behave appropriately and, to a lesser extent, on how competent the industry’s practitioners are deemed to be [9]. Since trust requires transparency, this must also be a high priority. Prioritising public trust is important for two reasons: not only is it a key driver of social licence, but it also creates a ‘halo effect’ that may influence the public’s view in times of crisis or challenge [105,106]. Any industry can unexpectedly reach a social licence ‘tipping point’ [103], and it is helpful if public trust has been established prior to such challenges [105,106]. Certainly, there is little question that if an organisation or industry has an unfavourable reputation, this will be a major liability in times of crisis [106]. However, a good reputation will not protect an organisation from the detrimental effects of a problem if it mounts an inappropriate response [106]. Whether an industry reaches the social licence tipping point largely depends on the way that it conducts its operations [9].

Another key principle that will help to optimise an activity’s social licence is the establishment of shared values (so-called ‘value similarity’) with stakeholders [9], followed by communication of these shared salient values. Along with transparency, this strategy is fundamental to establishment of trust [4]. Its importance is illustrated by work carried out in the food industry, which has shown that shared values are 3–5 times more important in building trust than demonstration of competence [103].

Positioning the industry as a front runner in the establishment of good practice is also helpful in terms of social licence retention [14]. This means much more than just meeting minimum standards [14] and requires the industry to be proactive in identifying and mitigating SLO threats, addressing welfare concerns, and reforming quickly in the light of new evidence [2]. Such reforms should consider both ethics and welfare and, where possible, should be grounded in science [9]. However, the absence of relevant research is not a reason to delay reform. Common sense and experience should play a role in any changes to rules and practice and, in conjunction with the precautionary principle, are sufficient to effect positive change within equestrianism whilst necessary research to support an evidence base is undertaken.

In natural resource industries, ensuring the safety of those who work in the industry, as well as those affected by it, is a key tenet of maintaining a social licence [14]. By its very nature, equestrianism is a risk sport, but it is important that all those involved continue to pursue avenues that minimise the risk of injury to riders and handlers. In conjunction with promoting environmental sustainability [107], maximising participant safety helps to demonstrate that an industry is socially responsible [108].

An industry’s social licence can be supported further by proactively engaging with the media, explaining how challenges have been met, publicising positive changes, and promoting champions of good practice [14]. In terms of company reputation and SLO, changing corporate behaviour or business practice in response to challenge significantly outperforms ‘business as usual’ across a range of industries [105]. Even taking positive action in an unrelated area (i.e., generating an indirect response to a challenge) is significantly more effective than doing nothing, although it is not as effective as a direct response [105].

**Figure 3:**
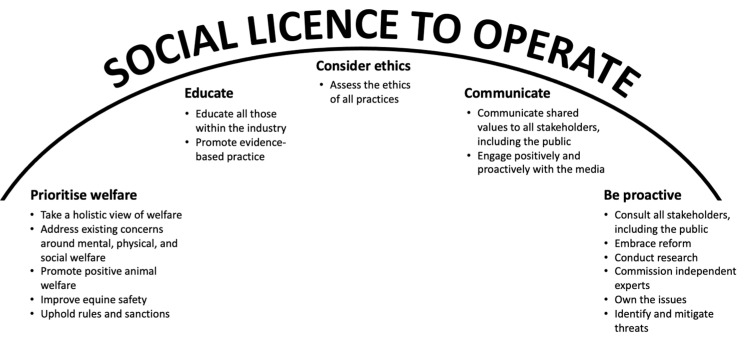
Actions that help to maintain an industry’s social licence to operate. The approach taken by an industry/group to its operations largely dictates the status of its social licence. Those industries that are able to pursue their activities with minimal formalised restrictions generally take a proactive approach to challenges, educate all stakeholders, and communicate well. If animals are involved in the activity, prioritisation of ethics and welfare is also important in maintenance of social licence. Industries that fail to take these steps invite regulation and/or legislation that controls or bans their activities.

The strategies outlined above all help to establish the legitimacy, credibility, and trust that are fundamental to the establishment of a healthy SLO [4,16] (Figure 3). Such an approach requires leaders with the courage to steer an industry in a direction that may not be popular in the short term, but that is likely to be beneficial in the long term [14]. Reflection on the fates of those animal-use activities that have lost their social licence suggests that brave action and a forward-thinking approach to equine welfare and equestrianism’s SLO will always be of benefit.

## 6. Positive Actions to Maintain Social Licence: Suggested Specific Actions for Equestrianism

We would like here to expand on two approaches to the maintenance of social licence which we suggest are of particular relevance to equestrianism: (i) engaging and communicating with stakeholders; (ii) adopting a holistic, evidence-based approach to the assessment of equine welfare and the ethics of equestrianism.

### 6.1. Engaging and Communicating with Stakeholders

Equestrianism’s stakeholders encompass a diverse range of organisations and individuals, including national and international sporting bodies, sponsors, commercial companies, officials, professional and amateur owners, trainers, riders/drivers, breeders, grooms, ancillary staff, and volunteers, as well as fans, spectators, and the wider public.

The key to optimal stakeholder engagement is honest, transparent, and collaborative consultation and communication [4,14,16,109]. If communication is to be truly collaborative, the sport must gain an understanding of the beliefs and desires of all stakeholders—including the sport’s critics—and engage with them in a constructive dialogue [2,16,110]. Undoubtedly, corresponding with the wider public and those who criticise equestrianism may be uncomfortable, but proactive engagement with stakeholders and the establishment of a shared vision for the future of the sport are key drivers of social licence [48]. As part of this, it is important to listen not only to external stakeholders, but also to the people who work with the horses, as the views of this sector are currently not always respected [111,112]. Given the historically disparate views of the wide range of stakeholders in equestrianism, achieving consensus about what constitutes ethically acceptable equestrianism in the twenty-first century will not be without challenges. However, collaborative consultation can result in a surprising degree of convergence across a diverse range of stakeholders [109]. Moreover, evidence from other industries shows that animal welfare groups will support those who engage in animal-use activities if they demonstrate transparency and a commitment to the optimisation of welfare [48]. To some within equestrianism, engagement with external stakeholders may create fear of a ‘mob rule’ scenario [113], in which change is driven by those who shout the most loudly, rather than being based in evidence. However, all stakeholders share a common interest in and responsibility for the optimisation of equine welfare. Preparedness by those within the sport to engage with external stakeholders in finding collaborative, evidence-driven ways of working together is part of that responsibility. 

In terms of communication, a number of overriding principles optimise maintenance of a healthy SLO. Firstly, as discussed above, communications that are based on shared values are more effective than those based solely on science [4]. Secondly, for animal-use activities, focussing on both ethics and animal welfare is key [9]. It is also important that any communications and messaging are underpinned by practice—in other words, equestrianism must ‘walk the talk’ [4], aligning its behaviour with the values and expectations of society [107]. It may also be helpful to adopt a ‘one welfare’ approach to strategy and communication [2], considering the effects of any change on equine welfare, human wellbeing, and conservation of the environment [114]. As part of this, promoting the benefits of the horse–human partnership to both horses and humans is likely to be beneficial [115]. Communication strategies that have been shown to be unhelpful include dismissing public concerns as reflecting lack of knowledge or understanding [2] and adopting a defensive or aggressive response to criticism [9]. Similarly, attempting to ‘educate’ the public in ‘the truth’ about equestrian sport is not effective, unless it displays shared values with all stakeholders [4].

### 6.2. The Value of an Evidence-Based and Holistic Approach to Equine Welfare

In terms of maintenance of social licence, value-based communications are substantially more effective than those based on science [4,9]. However, it is important that, as far as possible, any reforms adopted are evidence-based [9] if they are to have the desired positive impact on equine welfare. There should be clear acknowledgement that both ethical judgment and welfare science underpin the optimal use of animals [3,116], with a distinction made between the two. Equestrianism should therefore promote a culture whereby all stakeholders ask “Should I?” before they ask, ‘‘Can I?” [3,9,116]. Whilst acknowledging that the same evidence can lead to different conclusions, depending on the value system or ethical standpoint of the user [117], it is strongly recommended that a formal process to assess the ethics of various aspects of equestrian sports [118] be universally adopted. This would ensure, as much as possible, that the rationale underlying allowed practices has been examined from an ethical perspective. A pertinent example would be examination of the ethics behind the use of whips for ‘encouragement’ in racing [4].

There is a tendency within some animal-use industries to rely on technological and biomedical solutions to perceived welfare problems [111]. However, this alone is unlikely to improve public acceptance and trust [111]. In its place, the scientific study of animal welfare should be promoted, and the associated costs should be regarded as an investment in the future of the sport [2]. It is important that the ethicists and scientists who are involved in any research and monitoring are independent and that, as well as being scientifically credible, the resulting data are made publicly available [2]. In addition, the topics explored should encompass welfare in its entirety, as defined by welfare scientists, rather than being confined to those issues that the public currently deems to be important [111]. As part of this, it is helpful to listen to those who advocate reform of equestrian sports. These individuals may have a broader and more holistic understanding of welfare than those who work with horses and see more clearly what is necessary to protect the sport’s future—what insiders see as a problem of perception, outsiders may see as a problem of reality [111]. For example, there is evidence that some individuals within equestrianism have ‘normalised’ practices that, to many, are unacceptable [119], and that over-exposure to animals whose behaviour reflects compromised welfare has blinded people to the reality of these animals’ lives [120,121]. In addition, some sports practice ‘venue exceptionalism’ (e.g., use of a whip is acceptable at racetracks in a way that it would not be elsewhere) [4] and attitudes such as this are worthy of re-examination.

As discussed above, a number of equestrian sporting bodies have developed and published welfare strategies as part of a holistic approach to improving horses’ lives. This approach is likely to be helpful in maintaining the sport’s SLO [100]. However, its effectiveness will be maximised by a number of factors, including whether the strategy: was developed by individuals who are independent [2]; sets out minimum welfare standards, outlines ‘best practices’, and commits to constant, evidence-based review of the standards [122]; addresses the lifetime care of all equines [100]; promotes not only the absence of negative experiences for horses, but also the presence of positive experiences [100]; covers threats to equine welfare that may not be immediately obvious to the public, but that are potentially more problematic (e.g., lack of turnout, lack of opportunity to socialise with other equids, inhumane/suboptimal training practices) [100]; and avoids taking an anthropocentric approach to equine management and training [111,123]. The effectiveness of any welfare strategy in maintaining a sport’s SLO will also be influenced by the extent to which its recommendations are promoted and upheld [100].

If the beneficial effects of a welfare strategy on social licence are to be maximised, it should work hand-in-hand with the development of forward-thinking rules and codes of conduct relating to equine welfare. One potential example of this would be judging systems that reward good practices [100], such as optimal body condition or a positive affective state. To the fullest extent possible, all rules should be evidence-based, and sanctions for those who transgress should be meaningful [4]. Penalties for rule breaches should therefore be sufficiently robust to act as a deterrent and should be applied consistently and fairly [4], with independent oversight. Without doubt, inconsistency and ‘discretion’ relating to the imposition of penalties leads to loss of public trust [4], which is detrimental to social licence.

Finally, it is incumbent on equestrian sporting bodies to promote equine welfare science education to all those who are involved with horses [100] and to empower and support officials and judges at competitions to act when they see examples of poor welfare. Both the rules of the sport, and the knowledge of those within equestrianism, should reflect a welfare-focussed mindset that is based on the latest findings. It has been argued that many of those working at high levels within equine sports are not familiar with animal welfare science [111]. This may include the involvement of veterinarians, who should play a key role in safeguarding equine welfare [124,125,126]. It is also important that vets’ obligations towards the protection of equine welfare are understood by all stakeholders, including vets themselves [111].

## 7. Conclusions

The demise of a number of animal-use activities, including some that involve horses, makes it clear that the sustainability of equestrian sports depends on its ability to evolve and, in particular, to address issues relating to ethics and equine welfare. This review summarises strategies that have proved helpful in the maintenance of social licence in other industries, many of which are applicable to equestrianism. However, equine athletes’ situation is unique, even within the gamut of animal-use activities, and further consideration of equine-specific issues is necessary. Should exploration of these issues lead to the conclusion that, overall, the use of horses in sports is detrimental to their welfare, we would fully support the sport’s discontinuation. In the meantime, however, equine welfare is best supported by diligent attention to those welfare issues that are of concern to people from both inside and outside equestrianism.

Improvements in welfare are undoubtedly ongoing. Examples of best practices in equine care span numerous disciplines and countries, and many branches of equestrianism are taking positive steps to maintain their SLO. However, current threats to horse sport’s SLO are only likely to grow unless all those involved adopt a proactive approach to the issue, commit to the optimisation of equine welfare, and are able to justify their actions from an ethical standpoint. The establishment of trust and transparency are key, as is effective communication. In this regard, scientific underpinning of the sport’s practices is important but, to quote Theodore Roosevelt, “People don’t care how much you know until they know how much you care.” Communications should therefore be based primarily on values that are shared with all stakeholders (including the public) and should make clear all of the positive actions that equestrianism already implements to promote ethics-based practices and optimise equine welfare. Such communications must, however, also reflect the reality of practice, as unsubstantiated positive messaging (‘welfare washing’) will not result in a sustainable sport. For any industry or group, effort expended on earning and maintaining its SLO essentially represents enlightened self-interest, and the associated costs should be regarded as an investment in its future. Equestrianism can learn from the experience of other animal-use activities that have faced challenges to their SLO—both those that have survived and those that have not—as it maps its future.
